# 

**Published:** 1890-12-20

**Authors:** 


					ma HHW piuJIny Ull I Ill    ILIUM.     " I I -
made beef-tea, Ac., whereas had JOHNSTON'S BOvBIL been used in theirstead, UntfTIIDId, bnuucno* ?*.?
&?S?^5S!&?& w W <^5a=a THROUGHOUT
have gained strength IbL Id battle against -tup nuiTrn vninnnu
? the disease under v*w ^5 which they were THE UnlTcu KINbuURI.
suffering. I have no HA M iM.{ i hesitation to advise you
-tibe to your patients, to your oonyalesoents, to those of Jfflfcj &=-> 'Sh your clients who have mental exertions, to use Johnston's
whioh, in a concentrated form, contains a substantial L? ~) ^ tonic and a palatable food."?J. M. BEAUSOLIBL, M.D. ir.v
'No. 221. vol, IX.J Saturday,
December 20th, 1890.
[One Penny.

				

